# A synergistic antiproliferation effect of curcumin and docosahexaenoic acid in SK-BR-3 breast cancer cells: unique signaling not explained by the effects of either compound alone

**DOI:** 10.1186/1471-2407-11-149

**Published:** 2011-04-21

**Authors:** Jeffrey D Altenburg, Andrew A Bieberich, Colin Terry, Kevin A Harvey, Justin F VanHorn, Zhidong Xu, V Jo Davisson, Rafat A Siddiqui

**Affiliations:** 1 Cellular Biochemistry Laboratory, Methodist Research Institute, Indiana University Health, Indianapolis, Indiana, USA; 2 Laboratory for Chemical Biology and Drug Development Bindley Bioscience Center, Discovery Park, Purdue University, West Lafayette, Indiana, USA; 3 Department of Biology, Indiana University-Purdue University, Indianapolis, Indiana, USA; 4 Department of Medicine, Indiana University School of Medicine, Indianapolis, Indiana, USA

## Abstract

**Background:**

Breast cancer is a collection of diseases in which molecular phenotypes can act as both indicators and mediators of therapeutic strategy. Therefore, candidate therapeutics must be assessed in the context of multiple cell lines with known molecular phenotypes. Docosahexaenoic acid (DHA) and curcumin (CCM) are dietary compounds known to antagonize breast cancer cell proliferation. We report that these compounds in combination exert a variable antiproliferative effect across multiple breast cell lines, which is synergistic in SK-BR-3 cells and triggers cell signaling events not predicted by the activity of either compound alone.

**Methods:**

Dose response curves for CCM and DHA were generated for five breast cell lines. Effects of the DHA+ CCM combination on cell proliferation were evaluated using varying concentrations, at a fixed ratio, of CCM and DHA based on their individual ED_50_. Detection of synergy was performed using nonlinear regression of a sigmoid dose response model and Combination Index approaches. Cell molecular network responses were investigated through whole genome microarray analysis of transcript level changes. Gene expression results were validated by RT-PCR, and western blot analysis was performed for potential signaling mediators. Cellular curcumin uptake, with and without DHA, was analyzed via flow cytometry and HPLC.

**Results:**

CCM+DHA had an antiproliferative effect in SK-BR-3, MDA-MB-231, MDA-MB-361, MCF7 and MCF10AT cells. The effect was synergistic for SK-BR-3 (ER^- ^PR^- ^Her2^+^) relative to the two compounds individually. A whole genome microarray approach was used to investigate changes in gene expression for the synergistic effects of CCM+DHA in SK-BR-3 cells lines. CCM+DHA triggered transcript-level responses, in disease-relevant functional categories, that were largely non-overlapping with changes caused by CCM or DHA individually. Genes involved in cell cycle arrest, apoptosis, inhibition of metastasis, and cell adhesion were upregulated, whereas genes involved in cancer development and progression, metastasis, and cell cycle progression were downregulated. Cellular pools of PPARγ and phospho-p53 were increased by CCM+DHA relative to either compound alone. DHA enhanced cellular uptake of CCM in SK-BR-3 cells without significantly enhancing CCM uptake in other cell lines.

**Conclusions:**

The combination of DHA and CCM is potentially a dietary supplemental treatment for some breast cancers, likely dependent upon molecular phenotype. DHA enhancement of cellular curcumin uptake is one potential mechanism for observed synergy in SK-BR-3 cells; however, transcriptomic data show that the antiproliferation synergy accompanies many signaling events unique to the combined presence of the two compounds.

## Background

Breast cancer is now understood to be a collection of diseases characterized by malignant cells of different molecular phenotypes. Tumor subtypes are primarily categorized by expression of three cellular receptors: estrogen receptor (ER, HGNC gene symbol ESR1), progesterone receptor (PR, HGNC gene symbol PGR), and the epidermal growth factor receptor family member Her2/Neu (HGNC gene symbol ERBB2). Expression levels of all three cellular receptors are emerging as indicators of disease prognosis and criteria for determination of appropriate therapeutic regimen [1-5]. Because of this increased attention is being given to therapeutic strategies targeted to breast cancers based upon molecular subtypes [6-10]. Therefore, it should not be surprising that compounds exhibiting some utility as antiproliferation agents can show variable results when applied to cell lines of different ER/PR/Her2 phenotype. In fact, in an extensive characterization of the genetic and phenotypic variation among 51 breast cancer cell lines, Neve *et al. *even demonstrated variable potency of Trastuzumab among three Her2-overexpressing cell lines, with therapeutic response prediction later refined by post-hoc analysis of expression level for several other proteins and amplification of various chromosomal regions [11]. It stands to reason that investigation of anti-cancer dietary compounds will also benefit from a detailed look at interaction with cancer cell molecular phenotype. Only then will proper tailoring of dietary supplemental treatment to breast cancer subtype be facilitated. In this study we show that a combination of curcumin (CCM) and docosahexaenoic acid (DHA) results in variable antiproliferative effects across breast cancer cell lines of different molecular phenotype. For SK-BR-3, the cell line for which the effect is a synergistic improvement over either compound individually, the effect is accompanied by transcript and protein level changes that are not simply a combination of changes caused by either molecule alone.

DHA is an omega-3 fatty acid (22:6^Δ4,7,10,13,16,19^), part of a family of compounds reputed to possess many human health benefits, including anticancer properties [12]. Early epidemiological evidence strongly linked fish oil (rich in DHA and eicosapentaenoic acid) with lowered incidence of several types of cancer, including breast cancer [13-16]. In addition to epidemiological studies, dietary studies with mice and humans, combined with numerous tissue culture studies, have substantiated the beneficial role of DHA in breast cancer [17-20]. DHA induces apoptosis in cancer cells through multiple mechanisms [21-27]. DHA also induces cell cycle arrest through p21-mediated inhibition of cyclin-dependent kinase-2 (CDK2) activity and stimulates protein phosphatase activity [20,28]. In addition, transport of DHA and other fatty acids to the nucleus and binding to nuclear receptors such as peroxisome proliferator-activated receptor (PPAR) and retinoid X receptors (RXRs) have been reported. DHA is a general ligand of PPARs, but binds more selectively to RXR transcription factors [29]. RXRs form homo- or heterodimers with PPAR and other nuclear hormone receptor super-families that include receptors for steroids, thyroid hormones, retinoic acid and vitamin D. RXRs are in turn reported to regulate p21 expression in breast cancer MDA-MB-231 cells [30]. These recent developments suggest that DHA may also play a role in attenuation of breast cancer growth through a PPARγ/RXR-mediated mechanism. Finally, DHA down regulates CXCR4, indicating ability to reduce the metastatic potential of breast cancer cells by decreasing the surface expression of a pro-migratory molecule [31].

CCM [1,7-bis(4-hydroxy-3-methoxy phenyl) -1,6-heptadiene-3,5-dione] is a biphenyl compound naturally concentrated in the rhizome of the herb *Curcuma longa*, commonly known as *turmeric *in English, *haldi *in Hindi, and *ukon *in Japanese. CCM has been used in Asian medicine for over 3,000 years [32], and possesses a wide range of pharmacological activities including anti-inflammatory, anticancer, antioxidant, wound healing, and antimicrobial effects [33]. The pharmacology and putative anticancer properties of CCM have been extensively reviewed [34-38]. Preclinical studies have revealed chemopreventive potential of CCM for several cancers, including colon [39,40], duodenal [41], stomach [42], prostate [43], and breast [44]. CCM has been shown to block each step in the carcinogenesis process, namely tumor initiation, promotion, and progression [45]. CCM acts on multiple targets and inhibits activation of key cell signaling mediators, including NFκB (NFκB), AP-1, Cox-2, MMP9, PKC, and EGFR [38]. CCM dramatically induces transcription of PPARγ and activates PPARγ in hepatic stellate cells [39]. CCM regulates p21 expression through a p53-dependent pathway in several cellular models, including breast cancer cells [44,46-49]. CCM's antiproliferative effects, specifically on breast cancer cells, have been linked in multiple ways to the induction of apoptosis. For example, downregulation of c-Jun N-terminal kinase (JNK), upregulation of BAX as an effector of p53, downregulation of Bcl-2, inhibition of Akt/PKB, and generation of reactive oxygen species (ROS) have all been observed in breast cancer derived cell lines exposed to curcumin [23,50-52].

It is clear that DHA and CCM independently have biological activities that warrant development for therapeutic purposes, and combinations of the two have even been reported to exert synergistic effects against colon cancer inflammation and growth of pancreatic tumor xenografts [53,54]. However, productive application of these two compounds in a breast cancer context requires: (1) comparison of cell lines representing distinct molecular phenotypes associated with disease subclasses, (2) quantitative methods for detection of synergy versus additive effects upon cell proliferation, and (3) molecular characterization of cellular response sufficient to show whether any detected synergy is based upon novel mechanisms versus a straightforward merger of effects expected of each molecule individually. Accordingly, this study included five breast cell lines covering distinct cellular receptor expression phenotypes: SK-BR-3 (ER^- ^PR^- ^Her2^+^), MDA-MB-231 (ER^- ^PR^- ^Her2^-^), MDA-MB-361 (ER^+ ^PR^- ^Her2^+^), MCF7 (ER^+ ^PR^+ ^Her2^-^) and MCF10AT (ER^+^, PR isoform B but not A, Her2 variable) [11,55-58]. Across these cell lines, the antiproliferation effects of CCM, DHA, and a CCM+DHA combination were assessed quantitatively using methods designed specifically to detect the presence of synergistic versus additive or subadditive action. Detection of antiproliferation synergy for CCM+DHA within the SK-BR-3 cell line was followed by transcript analysis using the Agilent Whole Human Genome Microarray 4 × 44 K platform, demonstrating a broad gene regulatory response across several functional categories that have little in common with transcript level changes caused by CCM or DHA alone. Two protein level phenomena that could be expected from CCM and DHA individually, PPARγ expression and p53 phosphorylation, were both increased in the presence of CCM+DHA over levels observed for either compound alone. Finally, in addition to the combination causing novel intracellular molecular responses, DHA directly enhanced cellular uptake of CCM as demonstrated by flow cytometry and HPLC techniques. This suggests an added benefit of the combination in that, while DHA can achieve fairly high systemic concentration in humans, CCM on its own has limited bioavailability.

## Methods

### Cell lines and reagents

All cell lines were obtained from the American Type Culture Collections (ATCC; Manassas, VA) unless otherwise noted. MDA-MB-231, MDA-MB-361, and MCF7 cells were maintained in Dulbecco's modified eagle medium (DMEM; Invitrogen; Carlsbad, CA) supplemented with penicillin (100 units/ml), streptomycin (100 μg/ml) and 10% FBS. SK-BR-3 cells were maintained in McCoy's 5A medium (ATCC) supplemented with penicillin (100 units/ml), streptomycin (100 μg/ml) and 10% FBS. MCF10AT cells were purchased from Barbara Ann Karmanos Cancer Institute (Detroit, MI) and maintained in DMEM/F12 (Invitrogen; Carlsbad, CA) supplemented with penicillin (100 units/ml), streptomycin (100 μg/ml), 5% horse serum, insulin (10 μg/ml), epidermal growth factor (20 ng/ml), hydrocortisone (0.5 μg/ml), and cholera toxin (100 ng/ml). These supplemented media are referred to as complete media. All cell cultures were incubated in a humidified incubator at 37°C and 5% CO_2_. The pan-caspase inhibitor Z-VAD-FMK was purchased from Biovision (Mountain View, CA). DHA (NuChek Prep, Inc., Elysian, MN) was diluted in 100% ethanol to make 50 mM stock solutions. CCM (Sigma Aldrich, St. Louis, MO) was dissolved in DMSO to make 50 mM stock solutions. Stock solutions of DHA and CCM were further diluted in respective media prior to cell treatment. Final concentration of ethanol or DMSO in treated cells was less than 0.1%.

### Proliferation assays

Cells were seeded into 96 well plates (5 × 10^3^/well) in complete medium one day prior to treatment. For MDA-MB-231, MDA-MB-361, and MCF7, treatments with DHA, CCM, or CCM+DHA were done in DMEM supplemented with 2% FBS. For SK-BR-3, treatments were done in McCoy's 5A medium supplemented with 2% FBS. For MCF10AT, treatments were done in complete medium. All cells were treated for 24 hours at 37°C/5% CO_2 _with indicated concentrations of DHA,CCM or CCM+DHA along with cells treated with similar concentrations of ethanol and/or DMSO (less than 0.1%) as matching vehicle controls. Proliferation was analyzed using WST-1 assays in accordance with the manufacturer's instructions (Roche Biosciences, Indianapolis, IN).

### Analysis of synergy

Proliferation results were analyzed as previously described [59] with some modification. Pilot assays were carried out individually for DHA and CCM with each cell line in order to determine ED_50 _for each reagent (Table 1). Using the ratio of the ED_50_s, the proportion of each compound needed in a combination dose was calculated with the constraint that each compound account for 50% of the combination's potency. As described by Tallarida, for each observed percent effect in the individual dose response curves, a theoretical additive dose was found [59]. Experiments were then carried out using the fixed ratio combination of DHA and CCM at a variety of different doses. For both individual compounds, the actual combination, and the theoretical additive doses, dose response curves were fit using GraphPad Prism (GraphPad Software Inc., La Jolla, CA). For each, a four-parameter dose response curve was fit using non-linear regression [60]. To evaluate the potential synergy between the compounds, the least squares fit line for the theoretical additive curve and the actual CCM+DHA mixture were plotted on the same axes, and the two curves were compared using parameter estimates and their respective 95% confidence intervals. If the actual combination had a much different effect than expected, it was indicated by either a statistically significant shift in the ED_50 _or hillslope.

**Table 1:** ED_50 _values for DHA and curcumin effects on breast cancer cell lines.

			**Combination**
	**Initial ED**_**50 **_**(μM)**^**a**^		**Ratio (μM)**
	
**Cell Line**	**DHA**	**CCM**	**DHA:CCM**
	
MCF7	56.5 ± 8.2	28.0 ± 4.2	55:30
MCF10AT	93.2 ± 5.5	45.8 ± 2.5	95:45
MDA-MB-231	36.7 ± 6.5	34.7 ± 10.4	35:35
SK-BR-3	63.8 ± 1.5	39.2 ± 1.8	60:40
MDA-MB-361	52.5 ± 9.5	23.0 ± 4.2	50:25

^a^Initial ED_50 _values calculated as mean ± SD concentration required for 50% inhibition of proliferation from two independent triplicate assays.

### Analysis of synergy by combination index (CI)

The Loewe additivity model was used as a second method of analyzing the interaction between DHA and CCM [61]. The interaction between the compounds is reported as the combination index in the following equation:

In the equation, d1 and d2 represent the concentrations of the compounds in combination required to achieve *x *effect. Dx,1 and Dx,2 represent the concentrations of the same compounds individually that would quantitatively achieve the same *x *effect. A CI < 1.0 indicates that the combination is synergistic, and a CI > 1.0 indicates an antagonistic interaction. The combination indexes for this study were determined by using concentrations corresponding to the ED_50 _of dose response curves for DHA, CCM or CCM+DHA.

### Cell culture and treatments for whole human genome transcript level analysis

SK-BR-3 cells were plated in 6 well plates (2.5 × 10^5^/well) in complete medium overnight prior to treatment. Cells were treated with vehicle or with 30 μM DHA, 30 μM CCM, or CCM+DHA (12 μM CCM + 18 μM DHA) for 24 hours with nine replicates per treatment. Following treatment, the cells were washed three times with PBS and suspended in 500 μl Trizol Reagent (Roche Biosciences; Indianapolis, IN). The suspended cells were pooled in sets of three, resulting in triplicates for each condition. The samples were frozen at -80°C.

### Transcript level data collection and pre-processing

Cells in Trizol were shipped on dry ice to Miltenyi Biotec Genomics Services (MBGS) for analysis via a one-color Agilent Whole Human Genome Oligo Microarray platform. RNA processing, array hybridization, image collection and data processing were executed according to protocols described in the MBGS Service Report. Briefly, total RNA was isolated using a standard Trizol protocol and evaluated for quality on an Agilent 2100 Bioanalyzer platform. Cy3-labeled cRNA was produced using the Agilent Low RNA Input Linear Amp Kit (Agilent Technologies) following the manufacturer's protocol. Yields of cRNA and the dye-incorporation rate were measured with the ND-1000 Spectrophotometer (NanoDrop Technologies). The hybridization procedure was performed according to the Agilent 60-mer oligo microarray processing protocol using the Agilent Gene Expression Hybridization Kit (Agilent Technologies) and Agilent's recommended hybridization chamber and oven. Microarray Cy3 images were read and pre-processed for background subtraction using Agilent's Feature Extraction Image Analysis Software (FES). Inter-chip scaling and differential gene expression calls were produced by analyzing FES-produced feature signal intensities with Rosetta Resolver (Rosetta Biosoftware). For each chip feature, significance of difference between control and experimental conditions was tested by applying an error model-based hypothesis test to all control replicates as a group versus each treatment replicate individually. Theory and validation of this method are described in Weng *et al.*, 2006 [62]. The error model-based test results in a P value associated with the difference observed for each feature; difference values are reported as both log10 ratio and fold change of experimental signal intensity over control signal intensity.

### Processing of preselected candidate gene lists and gene ratio lists from MBGS

Hypothesis tests from Rosetta Resolver were reported for all array features under each treatment condition; these results were further sorted by MBGS to produce files containing preselected candidate gene lists (PCGL) for all three replicates of each treatment condition. Each PCGL includes all array features returning a fold change ≥ 2 (up or down) with an associated P value ≤ 0.01. This does not unambiguously resolve all responses on a gene-by-gene basis as individual genes may be represented by multiple features within the Agilent array and that some individual genes could be represented by multiple features that returned contradictory responses. Therefore, the process of isolating genes with unambiguous transcript level responses required further logical filtering of the Rosetta Resolver results.

PCGL results for each experimental condition were filtered to return features showing fold change in the same direction in all three replicates. For these features, fold change and P value were averaged across the three replicates. All P values are ≤ 0.01, but for cases in which a gene is represented by multiple features responding in the same direction we used mean P value as a decision rule for which fold change value to attribute to the corresponding gene. These features were then sorted according to gene identity. Genes were subsequently filtered using a conditional formula that removed genes with contradictory features and, within each gene, returned fold change values associated with features exhibiting the smallest mean P value.

We then constructed matrices of fold change values, allowing comparison of the responses of individual genes among treatments for the purpose of heatmap generation. Any gene that responded significantly to only one or two treatments required retrieving fold change values < 2.0 from the whole-array gene ratio files. To do this, we assembled a cumulative list of Gene IDs including all genes that responded with fold change ≥ 2.0, P ≤ 0.01, in at least one treatment. For a specific treatment, genes showing significant response were removed from the cumulative list, and remaining Gene IDs were used to query the whole-array gene ratio list for that treatment. In order to do this, gene ratio files from the three replicates of a treatment were combined, and means were calculated for the ratios and P values of all features. The set of gene IDs was then used to extract relevant feature data from the set of averaged feature data. Finally, a set of fold change values and P values was assembled, for a treatment condition, showing both genes that responded to that treatment and values for genes that did not have significant responses ≥ 2.0 in that treatment, but did in at least one of the other two treatments. This set of results supplied the values used to create the heatmap (see results section). Examples of the spreadsheets used to process the microarray data are available in the supplemental files (Additional file 1, 2, 3, 4, 5).

### Reverse transcript polymerase chain reaction

SK-BR-3 cells were plated in 6 well plates (2.5 × 10^5^/well) in complete medium overnight prior to treatment. Cells were treated with vehicle or with 30 μM DHA, 30 μM CCM, or CCM+DHA (12 μM CCM + 18 μM DHA) for 24 hours with nine replicates per treatment. Following treatment, the cells were washed three times with PBS and trypsinized. The cells were pooled in sets of three, resulting in triplicates for each condition. RNA was isolated using the RNeasy mini kit (Qiagen) according to manufacturer protocol. Complementary DNA was generated using Superscript III reverse transcriptase (Invitrogen) according to manufacturer protocol. PCR reactions were performed with 1 minute, 94°C, followed by 30 cycles of [1 minute, 94°C; 1 minute, 56°C; 1 minute, 72°C] followed by 5 minutes, 72°C. Primers for PCR reactions are listed in table 2. PCR products were visualized on 1% agarose gels. 15s ribosomal RNA was used as a loading control.

**Table 2:** Primers for PCR reactions

Gene	Forward primer	Reverse primer
CYP1A1	5'-ggactttaacccctacaggtatgt-3'	5'-ggatctttctctgtaccctggggtt-3'
CYP1A2	5'-cagaatgccctcaacaccttctccatcg-3'	5'-gtgatgtcccggacactgttcttg-3'
CYP1B1	5'-gagaacgtaccggccactatcact-3'	5'-gttaggccacttcagtgggtcatgat-3'
SERPINB5	5'-cttgcctgttccttttccac-3'	5'-tggagagtttgaccttggca-3'
CXCR4	5'-ggtggtctatgttggcgtct-3'	5'-tggagtgtgacagcttggag-3'
15s rRNA	5'-ttccgcaagttcacctacc-3'	5'-cgggccggccatgctttacg-3'

### Western blot analysis

SK-BR-3 cells were seeded into 6 well plates (2.5 × 10^5^/well) in complete medium one day prior to treatment. Following 24 hour treatments with 30 μM DHA, 30 μM CCM, or a mixture containing 12 μM CCM and 18 μM DHA, the cells were lysed with RIPA lysis buffer containing pervanadate (200 μM), NaF (1 mM), diisopropyl fluorophosphates (DIFP), and protease inhibitors (Roche Biosciences, Indianapolis, IN). Protein concentrations were normalized using BCA reagent according to the manufacturer's protocol (Pierce, Rockford, IL). SDS-PAGE membranes were probed with antibodies to p21, p53 and phospho-p53 (Cell Signaling Technologies, Danvers, MA), PPARγ and GAPDH (Santa Cruz Biotechnologies, Santa Cruz, CA).

### Curcumin uptake

The ability of each cell line to absorb CCM was quantified using flow cytometry. Cells were treated for 24 hours with escalating concentrations of CCM or combinations of DHA and CCM in McCoy's 5A with 2% FBS at indicated concentrations. Cells were then trypsinized and washed three times with cold PBS. Because CCM exhibits a green fluorescent signal [63,64], the cells were analyzed using the FL1 channel of a FACSCalibur flow cytometer (Becton Dickinson; Franklin Lakes, NJ) equipped with an air-cooled argon laser emitting at 488 nm wavelength. Fluorescence was detected through a 575 ± 26 nm band pass filter and quantified using CellQuest Software (Becton Dickinson; Franklin Lakes, NJ). Quantification results are presented as percent increase of the mean fluorescence intensity of the CCM treated samples, compared to untreated controls in triplicate assays, using gated cell populations that exclude dead cells and cellular debris.

### Quantification of curcumin uptake by HPLC

Quantification of CCM uptake by HPLC was performed to validate the flow cytometry method. Confluent cells in T75 flasks were treated with 20 μM CCM for 24 hours. Following treatment, the cells were washed three times with cold PBS and lysed with RIPA buffer. The lysates were sonicated for 5 seconds. Cell lysate (200 μl) and 20 μl internal standard solution (R6G in ethanol, 300 μg/ml) were added to a pyrex glass tube. Chloroform containing 0.05% BHT (7 ml), 3.5 ml methanol containing 0.05% BHT and 1.7 ml 0.5 M KOAC-HAC (1:1) buffer were added to the tube, vortexed for 1 minute, and centrifuged at 900 × g for 10 min. The chloroform layer was harvested and dried under nitrogen gas flow. The residue was dissolved in 200 μl ethanol and transferred to an HPLC sample vial. CCM uptake was analyzed by a reversed-phase HPLC method using a Shimadzu LC-20AT HPLC system equipped with a multi-wavelength diode array detector (DAD), a SIL-20AC_HT _autosampler, and an Ascentis^® ^C18 column (4.6 × 250 mm, 5 μm) (Supelco, Sigma-Aldrich; St. Louis, MO). A gradient mobile phase composed of 45% acetonitrile (0.1% TFA)-55% water (0.1% TFA) to 100% acetonitrile (0.1% TFA) was used. The flow rate was 1.0 ml/min and the detection wavelengths were 426 nm and 528 nm, respectively, for CCM and rhodamine 6G (internal standard). The CCM peak was identified by comparing to the reference standard, and the quantification of the CCM was preceded with an external standard curve combined with internal standard technology. The concentration of the CCM was normalized based on the protein concentration of the cell lysate.

### Statistics

Statistical analysis of data is described earlier for each specific experiment. Otherwise all comparisons were analyzed by ANOVA. P values less than 0.05 were considered significant. Triplicate experiments were repeated at least three times.

## Results

### DHA and curcumin exert synergistic anti-proliferative effects on SK-BR-3 cells

**Figure 1:**
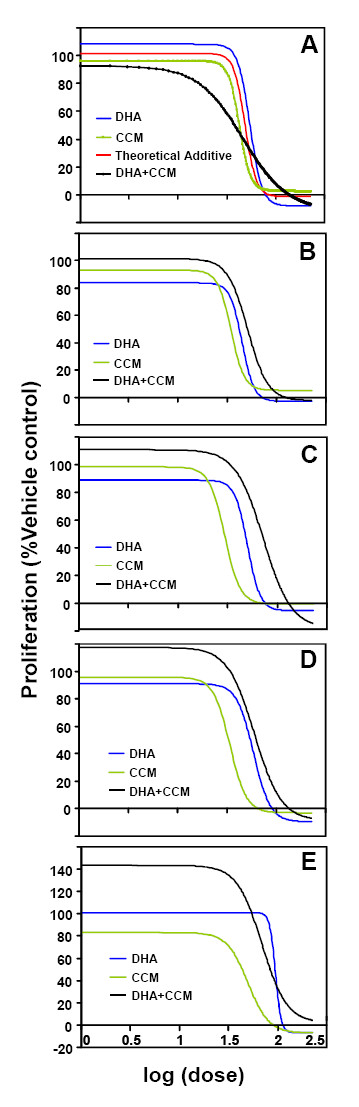
**The effects of DHA and CCM on breast cancer cell line proliferation**. SK-BR-3 (A), MDA-MB-231 (B), MDA-MB-361 (C), MCF7 (D), and MCF10AT (E) cell lines were treated for 24 hours with escalating doses of DHA (blue line), CCM (green line), or a 2:3 ratio of CCM+DHA (black line). A theoretical additive curve (red line (A)) was generated based on the curves for the individual compounds. Proliferation was measured with the WST-1 assay according to manufacturer protocol. Nonlinear regression of sigmoid dose-response model was performed with GraphPad Prism software. Results represent combinations of at least three triplicate experiments.

**Figure 2:**
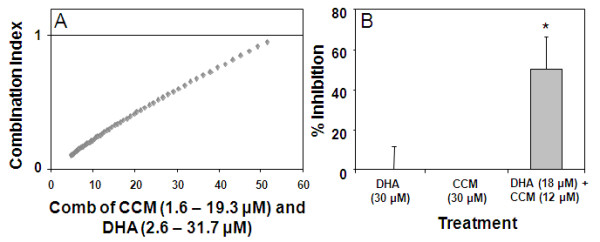
**The synergistic effect of CCM+DHA on SK-BR-3 proliferation**. (A) Combination index (CI) calculated for the SK-BR-3 cell proliferation. (B) Direct comparisons of 30 μM treatments with CCM and DHA individually with 30 μM treatment using the 2:3 ratio CCM+DHA combination. P < 0.05 for three triplicate experiments.

Initial cell proliferation assays were performed with dilution series (0-100 μM) of DHA and CCM using four breast cancer cell lines and one line (MCF10AT) representing 'premalignancy', each with a unique pattern of cellular receptor expression: SK-BR-3 (ER^- ^PR^- ^Her2^+^), MDA-MB-231 (ER^- ^PR^- ^Her2^-^), MDA-MB-361 (ER^+ ^PR^- ^Her2^+^), MCF7 (ER^+ ^PR^+ ^Her2^-^) and MCF10AT (ER^+^, PR isoform B but not A, Her2 variable). ED_50 _values were determined for DHA and CCM, for each cell line, and presented in Table 1. A combination of CCM+DHA (with proportions of DHA and CCM derived from their ED_50 _values) was used to measure the antiproliferation effect for each cell line at different doses (0-100 μM) as described by Tallarida [59]. The dose response curve for CCM+DHA was then compared to the theoretical additive dose response curve to determine if the combination of CCM+DHA resulted in a synergistic effect. It can be seen that the combination of CCM+DHA (2:3 ratio) when used below 50 μM exerted a synergistic effect only in the SK-BR-3 breast cancer cell line (Figure 1A). While there was no substantial difference in ED_50 _values, there was a significant difference in the hillslopes [(-7.6; 95% CI (-10.2, -5.1) for the theoretical additive curve and -1.9; 95% CI (-2.8, -1.0) for the actual mixture)]. This indicates that the span of doses where the actual combination of CCM+DHA is effective is much greater than expected. The synergism between DHA and CCM disappeared at higher doses, though a dose-dependent antiproliferative effect was still present. CCM+DHA also affected the MDA-MB-231, MDA-MB-361, MCF7, and MCF10AT cell lines, but subadditive to additive results were observed at all combinations tested (Figure 1B-E). Extending the treatments to 48 hours had no significant effect on the synergistic or subadditive status of each cell line (data not shown). The synergistic effect of CCM+DHA on SK-BR-3 cells was further confirmed using an alternative approach of calculating the Combination Index. The combination of CCM+DHA at concentrations below 50 μM had a Combination Index < 1, indicating synergism between two compounds (Figure 2A). We further experimentally compared antiproliferative effects of the combination with each compound individually using an optimal concentration (30 μM) and found that neither DHA or CCM were effective in inhibiting cell growth, whereas 30 μM of the combination (18 μM DHA + 12 μM CCM) significantly inhibited SK-BR-3 cell growth (Figure 2B).

### DHA + Curcumin regulatory effects on genes of disease-relevant functional groups

Transcript level changes were assessed for three replicate SK-BR-3 cell populations for each of three 24 hour treatment conditions: CCM (30 μM), DHA (30 μM) and CCM + DHA (12 μM + 18 μM). The Agilent whole human genome microarray results for each of the three treatment conditions were compared with transcript levels for SK-BR-3 cells left untreated in growth medium for 24 hours. As described in Methods, Rosetta Resolver was used to assign P values to individual chip elements for differences between untreated and treated cells using an error model-based hypothesis test. All chip elements meeting the criteria of fold-change ≥ 2.0 (p < 0.01), in any experimental replicate, were further filtered to return only transcripts for which at least one chip element responded with 2-fold or greater change, in the same direction, p < 0.01, with no other chip element indicating a significant contradictory directional change, across all three replicates for a treatment. According to these criteria, the numbers of transcripts exhibiting significant change for each treatment are as follows: CCM, 8,817; DHA, 61; CCM+DHA, 1,449 (Additional file 6).

**Figure 3:**
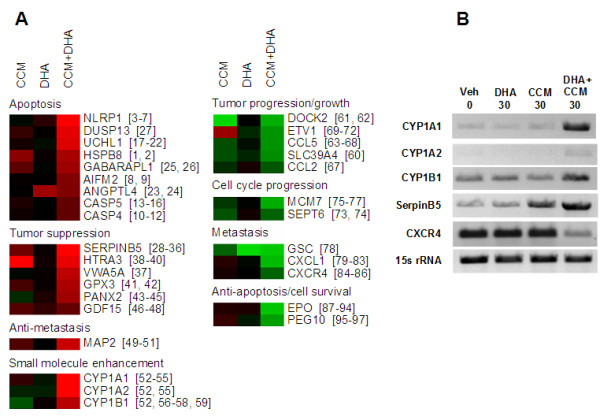
**Transcripts of functional importance relative to the synergistic antiproliferative effect of the curcumin/DHA combination**. (A) All genes are labeled according to current HGNC symbols. Numbers to the right of gene symbols indicate references in a separate transcript annotation bibliography (see Additional file 8). Fold-change and associated P values corresponding to this figure can be found in Additional file 9. Heatmap values are log_2_-transformed, normalized fluorescence ratios for untreated versus treated cells (see methods), with green indicating upregulation and red indicating downregulation relative to untreated SK-BR-3 cells. All responses shown for DHA+CCM were 2-fold or greater, p < 0.01, on three replicate arrays. For the purpose of visual comparison within a heatmap format, values for non-significant responses are mean normalized fluorescence ratios from three replicate arrays. For genes represented by more than one chip feature in the Agilent platform, mean normalized fluorescence ratio was retrieved for the feature producing the lowest mean P value. As such, some responses in this figure appear greater than 2-fold but were not significant according to the criterion p < 0.01. (B) RNA was isolated from SK-BR-3 cells treated with 30 μM DHA, μM CCM, or a mix of 12 μM CCM+18 μM DHA. RT-PCR was performed for selected genes in order to validate the microarray data. Results are representative of three separate experiments.

The most striking initial result was that CCM+DHA caused far fewer significant transcript level changes in SK-BR-3 cells than CCM alone, even though CCM+DHA had a greater antiproliferative effect. A cursory examination of the data also showed that some of the transcripts responding to CCM+DHA did not appear as significant responders in either of the other two treatments. Therefore, our strategy for an initial informatic analysis of these data was to rank CCM+DHA responders by magnitude of change, place a minimum cutoff at 5-fold (up or down), and annotate genes appearing within this set according to known disease-relevant functions. In addition, CCM+DHA responders below 5-fold were scanned for genes of known relevance to antiproliferation processes, such as caspases. Figure 3 illustrates the results in the form of a heatmap divided into functional blocks. This strategy recovered an interesting picture showing 31 functionally diverse transcript regulatory responses, many of which are unique to the CCM+DHA combination. We observed that genes involved in tumor progression/growth, cell cycle progression, metastasis, and anti-apoptosis/survival were synergistically downregulated by the combination, while genes involved in apoptosis, tumor suppression, and inhibition of metastasis were synergistically upregulated. Interestingly, we also found a group of cytochrome p450 genes (1A1, 1A2, 1B1), which are involved in enhancement of anti-cancer effects of small molecules, including some dietary compounds (see discussion below). Results from the microarray transcript analysis were further validated by RT-PCR for selected genes, and our results (Figure 3B) have validated changes observed within the microarray data. RT-PCR also confirmed that CYP1A1/CYP1A2 and CXCR4 did not respond to DHA or CCM alone, but their expression was stimulated or reduced, respectively, by combined DHA+CCM treatment (Figure 3B). Similarly, RT-PCR data also verified other directional changes indicating that the CCM effect on CYP1B1 was reversed by CCM+DHA, whereas SERPINB5 expression was enhanced following CCM+DHA treatment. While it is important to confirm expression levels of proteins directly before concluding specific signaling mechanisms, the genome-wide study of transcript and protein level correlation by Shankavaram *et al. *demonstrates predictive power of transcripts for protein levels across the NCI60 cell line panel[65]. Therefore, as a hypothesis building tool, transcript screens serve as valuable indicators of relevant network areas to study further.

SERPINB5 and CYP1A1/1A2/1B1 are transcripts of special functional interest. SERPINB5 produces a protein also known as maspin, and is thought to be a major downstream effector for the tumor suppression effect of Tamoxifen [66-68]; therefore, it is possible that CCM+DHA modulates a pathway overlapping that of Tamoxifen. CYP1A1/1A2/1B1 are members of the cytochrome P450 family. They are thought to enhance the anticancer effects of some small molecules, including dietary compounds, by metabolizing them into other structures with additional antiproliferative effects [69-72].

### CCM+DHA effects are mediated through an apoptotic process

**Figure 4:**
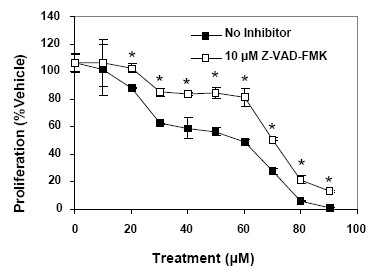
**CCM+DHA inhibit proliferation through a caspase-mediated process**. SK-BR-3 cells were pretreated for one hour with 20 μM Z-VAD-FMK, a pan-caspase inhibitor, prior to the addition of escalating doses of CCM+DHA (2:3 ratios). After 24 hour incubation, proliferation was analyzed with WST-1 reagent according to manufacturer protocol. Results are representative of three separate triplicate experiments. *P < 0.05 for Student's t-tests comparing the treatments with or without the pan-caspase inhibitor.

As shown above, our data clearly indicated an induction of genes involved in apoptosis. In order to further validate contribution of apoptosis to the reduction of cell proliferation during CCM+DHA treatment, we used a pan-caspase inhibitor (Z-VAD-FMK) to inhibit initiation of the apoptotic process. Data shown in Figure 4 indicate that CCM and DHA, in varying concentrations of a fixed 2:3 ratio, inhibited cell proliferation in a dose dependent manner (20-90 μM), and the pan-caspase inhibitor significantly reduced the effect of CCM+DHA at all concentrations tested.

### Protein level mediators of CCM+DHA synergistic effect

**Figure 5:**
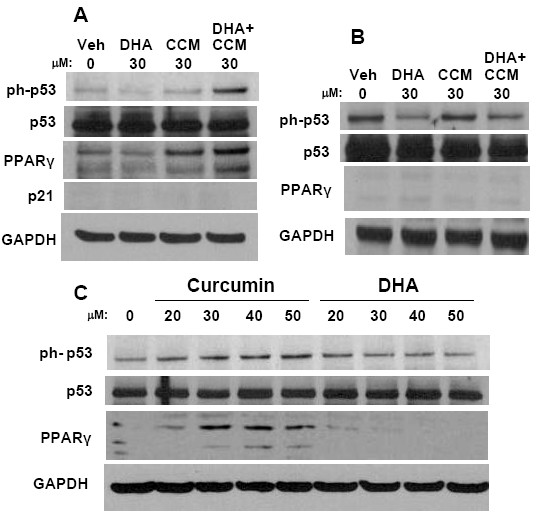
**Effects of CCM and DHA on p53 and PPARγ**. SK-BR-3 (A) and MCF-7 (B) cells were treated with 30 μM DHA, CCM, or the CCM+DHA combination (12 μM CCM and 18 μM DHA) for 24 hours in 6 well plates. (C) SK-BR-3 cells were treated with escalating doses of CCM or DHA individually. Cells were lysed and analyzed by SDS-PAGE. Blots were probed for phosphorylated p53, overall p53, p21, PPARγ, and GAPDH as indicated. Data are representative of three separate duplicate experiments.

One common mediator for DHA and CCM is the cell cycle regulator p21. In contrast to our prediction, we found that DHA and CCM alone or in combination do not increase p21 protein expression in SK-BR-3 cells (Figure 5A), but can increase p21 in the MDA-MB-231 cell line (data not shown). As mentioned in the introduction, CCM has been previously reported to affect p53 activity, and DHA is known to be a ligand for PPARγ; therefore, we investigated the roles of expression and activation for p53 and PPARγ by DHA, CCM or the combination relative to their synergistic growth inhibitory effects. We observed that treating SK-BR-3 cells with 30 μM CCM+DHA (2:3 ratio) resulted in increased phosphorylation of p53 and increased expression of PPARγ (Figure 5A). These increases were not detected when the cells were treated with 30 μM DHA alone, and the effects were lower with 30 μM CCM alone. Increases in p53 phosphorylation and PPARγ expression were not observed in MCF7 cells with identical treatments (Figure 5B), suggesting that the effects are specific for the SK-BR-3 cell line. When treated with a dilution series of either DHA or CCM alone, phosphorylation of p53 and upregulation of PPARγ only occurred with CCM treatment (Figure 5C), which suggests that the DHA portion of the combination enhanced CCM-mediated effects through the p53 and PPARγ pathway.

### Effects of DHA on curcumin uptake

**Figure 6:**
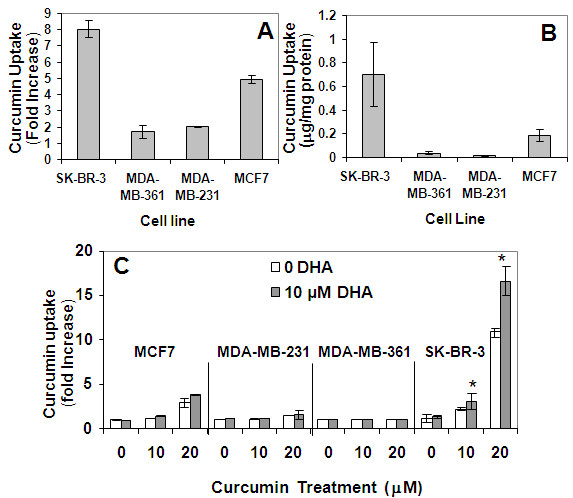
**Effect of DHA on CCM uptake**. Cells were treated with 20 μM CCM for 24 hours. CCM uptake was quantified by flow cytometry (A) in comparison with HPLC (B) as described in Materials and Methods. (C) SK-BR-3, MDA-MB-231, MDA-MB-361, MCF7, and MCF10AT cell lines were treated with escalating doses of CCM in the presence or absence of 10 μM DHA and analyzed by flow cytometry. Fold changes (A, C) were compared to respective cell line controls (without CCM or DHA). *P < 0.05 for Student's t-tests comparing the treatments with DHA to the treatments without DHA in three duplicate assays.

While the mechanism of CCM entry into cells is unknown, we have analyzed the differences among four breast cancer cell lines for ability to absorb CCM. We quantified CCM uptake by flow cytometry as CCM is known to fluoresce in the green band [63,64]. In order to validate the flow cytometry results, HPLC analysis was also performed concurrently with initial flow cytometry experiments (Figure 6A-B). The flow cytometry data match trends seen in the HPLC results, but with smaller dynamic range. SK-BR-3 cells treated with 20 μM CCM absorbed four-fold greater levels of CCM than the MCF7 cells and 10-fold greater levels than the MDA-MB-231 and MDA-MB-361 cells (Figure 6C). Combining DHA with CCM further enhances the CCM absorption of SK-BR-3 cells without significantly enhancing CCM uptake in the other cell lines (Figure 6C). This suggests that DHA enhancement of cellular permissiveness for CCM absorption is a potential mechanism for the increased antiproliferative effects. It should be noted that both HPLC and flow cytometry do not distinguish CCM taken into the cell from CCM that is bound to the cell surface. However, it is clear that the SK-BR-3 cells exhibit a higher level of CCM fluorescence than the other cell lines and that the fluorescence is increased in the presence of DHA. When viewed with the data describing antiproliferative effect and molecular level changes, it is unlikely that these data reflect only CCM on cell surfaces.

## Discussion

The idea that changes in diet or diet supplementation may improve the health of cancer patients or enhance the effectiveness of existing treatments is a compelling motivation for exploring the activities of dietary compounds. The translational process for such molecules benefits from a relative lack of toxic side effects and source material that is inexpensive and easily accessible relative to synthetic pharmaceuticals. Human diets can routinely encompass many biologically active small molecules, and evidence for synergy between dietary compounds is emerging [53,54,73]. In this study we have presented data demonstrating that at low concentrations, a combination of the omega-3 PUFA, DHA, and curcumin, a molecule found in turmeric, exerts a synergistic antiproliferative effect on the estrogen receptor negative, HER-2 positive SK-BR-3 breast cancer cell line. The degree of synergy decreased, relative to activity of each compound alone, as concentration of the CCM+DHA combination was increased, though total anti-proliferative effect continued to increase with concentration. This observation has also been made by others reporting synergy between DHA and CCM [53]. The fact that synergy is observable at low concentrations is of special interest in this case because while the antiproliferative effects of CCM alone may be more potent than those of DHA, CCM is known to be poorly absorbed and has been observed to remain under 2 μM in the serum and urine of human subjects receiving several grams per day [74]. Conversely, DHA can achieve plasma concentrations of approximately 200 μM in humans administered daily doses of oral DHA preparations over the course of a month [75]. These human subject data become especially interesting as we have demonstrated a DHA mediated enhancement of cellular CCM uptake. This means that when DHA and CCM are used in combination, the intracellular concentration of CCM achievable *in vivo *may not be limited to the concentration range previously observed. Careful, tissue-specific pharmacodynamics studies are needed to determine how our cell culture results can translate to a clinical setting, but the practical utility of this compound combination is promising.

As mentioned above, breast cancer is a collection of diseases with multiple phenotypes; therefore, one should not expect that a particular therapeutic agent will control every malignancy subtype. The fact that only the SK-BR-3 cell line was synergistically affected by DHA and CCM suggests specific breast cancer phenotype as an important factor for predicting efficacy. The ER^-^/Her2^+ ^phenotype is regarded as an aggressive tumorigenic phenotype. Our data suggest that molecular phenotype has specific implications for efficacy, and translation of these compounds into therapeutics will require extensive mechanistic studies. Beyond demonstrating the synergistic effect itself, the importance of this study lies in the demonstration that observed synergy is accompanied by cell signaling changes that are unique to the combination of the two compounds. A network state appears in which the aggregate of protein levels, protein states, and transcript levels are an emergent property of the combined presence of DHA and CCM.

The results shown in Figures 5 and 6 demonstrate that the synergistic effect of the CCM+DHA combination may in part be attributed to protein level effects described for CCM alone, but that are increased in the presence of DHA. CCM can increase the protein pool of PPARγ and increase phosphorylation of p53. Addition of DHA further increases both of these molecular effects and increases uptake of CCM by SK-BR-3 cells. This is interesting in that it fits a pattern observed for DHA in which it enhances the effects of other anti-cancer compounds: 5-fluorouracil on colon cancer cells [76], celecoxib on prostate cancer cells [77], and doxorubicin [78] in breast cancer. Our results, combined with previous studies, suggest that part of DHA's activity is to alter cellular permissiveness for uptake of several kinds of small organic molecules, possibly through alteration of membrane lipid composition. Uptake of CCM and DHA enhancement of this process were observed to be much stronger in SK-BR-3 cells relative to four other breast cancer cell lines, suggesting that the pharmacodynamics of the CCM+DHA combination may be dependent upon specific cancer phenotype, apart from the signaling changes that occur once the compounds actually enter a cell. Therefore, it is possible that the synergistic effect of CCM+DHA on SK-BR-3 cells is dependent specifically upon CCM uptake ability of this cell line, as opposed to its cellular receptor expression state, but further study of this distinction must account for the unique transcript-level regulation observed in the presence of CCM+DHA and not CCM alone. In this study we did not investigate the potential mechanism for CCM uptake by different breast cancer cell phenotypes, which is clearly a logical direction for our future studies.

Our microarray data suggest that cell cycle arrest and apoptosis include contributions from other events unique to the CCM+DHA combination upstream, downstream, or independent of PPARγ and phosphorylated p53. Two interaction networks were generated within the MetaCore (GeneGo) network analysis environment to illustrate all kinases documented as activators of p53 and all proteins documented to contribute to upregulation of PPARγ transcription (Additional file 7). From this it is clear that these two processes, while stimulated by CCM alone, can be upregulated through many alternate pathways. A 10-fold upregulation is seen for NLRP1, DUSP13 and UCHL1, all positively associated with apoptosis (see references in Additional file 8 for Figure 3) and all responding only to the CCM+DHA combination. Similar results are seen for genes associated with tumor suppression and MAP2, considered to be anti-metastatic. Downregulation is observed for genes associated with tumor progression, cell cycle progression, metastasis and anti-apoptosis/cell survival. Some of these were downregulated only when both DHA and CCM were applied to SK-BR-3 cells. Taken together, these results suggest multiple mechanisms in addition to enhanced CCM activity.

Two transcriptional responses of particular interest are CYP1B1 and SERPINB5. CYP1B1 was actually downregulated 2.4-fold by CCM alone, but this was reversed to 7.4-fold upregulation by the CCM+DHA combination (see Additional file 9 for Figure 3). DHA alone had no effect on this transcript. CYP1B1 is thought to contribute to the anti-proliferative effects of several dietary compounds by metabolizing them into products exhibiting additional cytotoxicity within cancer cells [69-72]. Presence of wild-type CYP1B1 was reported to be associated with protection against breast cancer in a population of Indian women [79]. SERPINB5, the protein product of which is also known as maspin, was upregulated 18.9-fold when SK-BR-3 cells were treated with CCM+DHA. Maspin is known to be upregulated by tamoxifen and is considered to be a downstream effector for the tumor suppression activity of tamoxifen [66-68]. It is, therefore, suggested that CCM+DHA induces an anticancer effect through a mechanism that is shared by tamoxifen. Furthermore, SK-BR-3 cells are ER-, raising the question of whether the CCM+DHA combination can contribute to overcoming tamoxifen resistance.

The anti-proliferative effect of CCM+DHA on SK-BR-3 cells is validated as a true synergy by molecular level characterization indicating that the emergent cell signaling network state is unique to the combined use of the two compounds. Transcript level responses are not an additive result of changes that would be predicted based upon activity of either compound alone. However, the functionally categorized heatmap blocks presented in Figure 3 are themselves only a beginning for the process of determining a mechanism of action. This preliminary analysis shows either transcript level changes that were strong in magnitude, for genes of some known functional description, or changes for which a direct connection with a process of interest is known, even if response was moderate. These transcriptional responses along with the synergistic antiproliferative effect demonstrated for SK-BR-3 cells are a compelling collection of disease relevant observations. However, the total genome-wide microarray data set requires network analysis on a scale beyond the scope of this study. Given the collection of strong transcript responses and the protein pool/activation responses of what can be considered two major signaling hubs (PPARγ and p53), the complete set of network nodes that mediate cellular response to CCM+DHA is likely large and inclusive of multiple pathways. Construction of a working model and subsequent experimental dissection of that model requires a modular, carefully annotated assembly of molecular responses into network neighborhoods in which both interactive relationships and transcriptional response values can be visualized. In this way, transcriptional responses and changes in protein states can be studied for patterns of causation that suggest much more specific mechanistic hypotheses than are apparent in a static representation of expression data such as a heatmap. Additionally, the observation of synergy in only one of five different breast cancer cell phenotypes suggests that signal paths critical to cell proliferation are not uniformly accessible to the CCM+DHA combination in all breast cancer phenotypes. Determining the network of interacting nodes responsible for the SK-BR-3 response to CCM+DHA should in turn reveal a set of signaling nodes that are candidates for being the root cause of phenotypic differences among breast cancer types.

## Conclusions

Our data suggest that combination of DHA and curcumin may provide a novel, effective dietary supplemental treatment for some breast cancer patients, with a likely dependency upon molecular phenotype. It is important to stress that the synergistic antiproliferative effects were only observed in the SK-BR-3 cell line. Analysis of other breast cancer cell lines representing the ER^- ^PR^- ^HER2^+ ^phenotype is required to confirm that the effect is related to cancer subtype and not solely a property of the SK-BR-3 cell line itself. In addition, there are breast cancer cell lines that match SK-BR-3 cells with respect to other characteristics (for example, expression of the luminal versus basal gene clusters) [11], and the possibility that a mechanistic explanation of synergy lies within these alternate characters is another immediate focus for extension of this work. DHA enhancement of cell permissiveness to curcumin uptake is one potential mechanism for the observed synergy. However, our microarray analysis suggests that some mechanistic pathways are triggered in the presence of the curcumin/DHA combination and do not appear when either compound is used alone, indicating that at least part of the observed synergy is an emergent property of the combination specifically. Although this study, as conducted in an *in vitro *system, has certain limitations, a set of unique genes was identified for the synergistic effect of DHA and CCM. Studies in an *in vivo *system are currently underway to further validate the findings of the present study.

## Abbreviations

DHA: docosahexaenoic acid; CCM: curcumin; PPAR: peroxisome proliferator-activated receptor; ER: estrogen receptor; PCGL: preselected candidate gene lists; MBGS: Miltenyi Biotec Genomics Services.

## Competing interests

The authors declare that they have no competing interests.

## Authors' contributions

JDA performed experiments, participated in the experimental design, and drafted the manuscript. AAB performed analysis of the microarray results and helped to draft the manuscript. CT participated in the design of experiments, performed statistical analysis and helped to draft the manuscript. KH performed immunoassays and participated in the experimental design. JFV performed immunoassays and HPLC experiments. ZX performed HPLC experiments, analyzed HPLC results, and helped to draft the manuscript. VJD participated in experimental design and helped to draft the manuscript. RAS conceived of the study, and participated in its design and coordination and helped to draft the manuscript. All authors read and approved the final manuscript.

## Pre-publication history

The pre-publication history for this paper can be accessed here:

http://www.biomedcentral.com/1471-2407/11/149/prepub

## Supplementary Material

Additional file 1 **Extracting three-replicate responsive genes from Miltenyi Biotec microarray data**. The Excel file "Supplementary Data-1.1.xlsx" demonstrates the first step in logical filtering of preselected candidate gene lists (PCGL) that are provided as pre-processed microarray data by Miltenyi Biotec. Each PCGL is a list of the microarray elements that returned a significant response within one replicate of a treatment (fold change ≥ 2 and P ≤ 0.01). Most transcripts (genes) are represented by more than one microarray element, and these do not always agree with each other, rendering the result for a gene ambiguous. Also, a single microarray address may not return the same result across all three replicates. Therefore, logical filters were implemented as conditional statements in Excel to compile a list, for each treatment, of only those genes that respond significantly in a consistent direction across three treatment replicates. Step one for the Zadd treatment (CCM+DHA) is presented as an example. Click here for file

Additional file 2 **Extracting identities of genes that did not respond to one treatment but did respond to another treatment**. In order to assemble the heatmap in Figure 3, it was necessary for each gene to have a cell value across all three treatments, including those instances in which a particular gene had no response to a given treatment. The Excel file "Supplementary Data-1.2.xlsx" demonstrates, for the Zadd treatment, how to assemble the list of microarray elements that were isolated as three-replicate responders for either of the other two treatments, but that returned no significant response for all three replicates of the Zadd treatment. This list is used in the next step to query the raw data for Zadd in order to collect fold-change values for these elements. Click here for file

Additional file 3 **Extracting ratio, fold-change and P values for genes that did not respond to one treatment but did respond to another treatment**. The microarray element identities compiled in Supplementary Data-1.2 were then used in "Supplementary Data-1.3.xlsx" to query the gene ratio lists (comprehensive data for all microarray elements) for each replicate of the Zadd treatment. Results for each element are compiled into a single final data set sorted by element ID. Click here for file

Additional file 4 **Mean ratio, fold-change and P values for "ALL not Zadd" microarray element list, checked against three-replicate responders for Zadd**. In the Excel file "Supplementary Data 1.4.xlsx", mean ratio, mean fold-change and mean P values are calculated for all microarray elements compiled in Supplementary Data-1.2. These are then sorted by gene ID, and each gene is represented by the microarray element returning the lowest mean P value. This list of genes is then checked against Zadd three-replicate responders in order to catch disagreement among microarray elements. Click here for file

Additional file 5 **Final set of Zadd gene responses for all genes that responded to any of the three treatments**. This final data set contains treatment/control ratios, fold-change values and P values, from Zadd treatment replicates, for all transcripts that responded ≥ 2-fold, P ≤ 0.01, in any of the three treatments. For any transcript that was not a three-replicate responder in the Zadd treatment, values were derived as described in Supplementary Data 1.1-1.4. The set of values in Excel file "Supplementary Data 1.5.xlsx" served as the source of transcript response values for Zadd in Figure 3. Transcript responses used in Figure 3 for each of the other two treatments, CCM and DHA, were derived using the same workflow. Click here for file

Additional file 6 **Distribution of fold change by treatment for whole human genome transcript analysis**. This figure represents the raw distribution of fold change magnitude for all transcripts that exhibited fold change ≥ 2.0 (p < 0.01) in all three replicates of at least one treatment. Click here for file

Additional file 7 **Possible routes to phosphorylation of p53 and upregulated transcription of PPARγ**. p53 and PPARγ were each used as single beginning nodes around which to build one-step expansion networks within MetaCore™ version 6.3 (GeneGo). Click here for file

Additional file 8 **Literature annotation for transcript functional categories in Figure **3. Numbered references included in this file match reference numbers appearing to the right of each HGNC symbol in Figure 3. Click here for file

Additional file 9 **Data represented by Figure **3. Fold change values and associated P-values for transcripts represented in Figure 3. Click here for file
