# Supplementary material for: A synergistic antiproliferation effect of curcumin and docosahexaenoic acid in SK-BR-3 breast cancer cells: unique signaling not explained by the effects of either compound alone
Source: BMC Cancer. 2011 Apr 21;11:149. doi: 10.1186/1471-2407-11-149 (PMC3111403; doi:10.1186/1471-2407-11-149)
Supplement: Additional file 6 — Distribution of fold change by treatment for whole human genome transcript analysis. This figure represents the raw distribution of fold change magnitude for all transcripts that exhibited fold change ≥ 2.0 (p < 0.01) in all three replicates of at least one treatment. [file 1471-2407-11-149-S6.PDF]

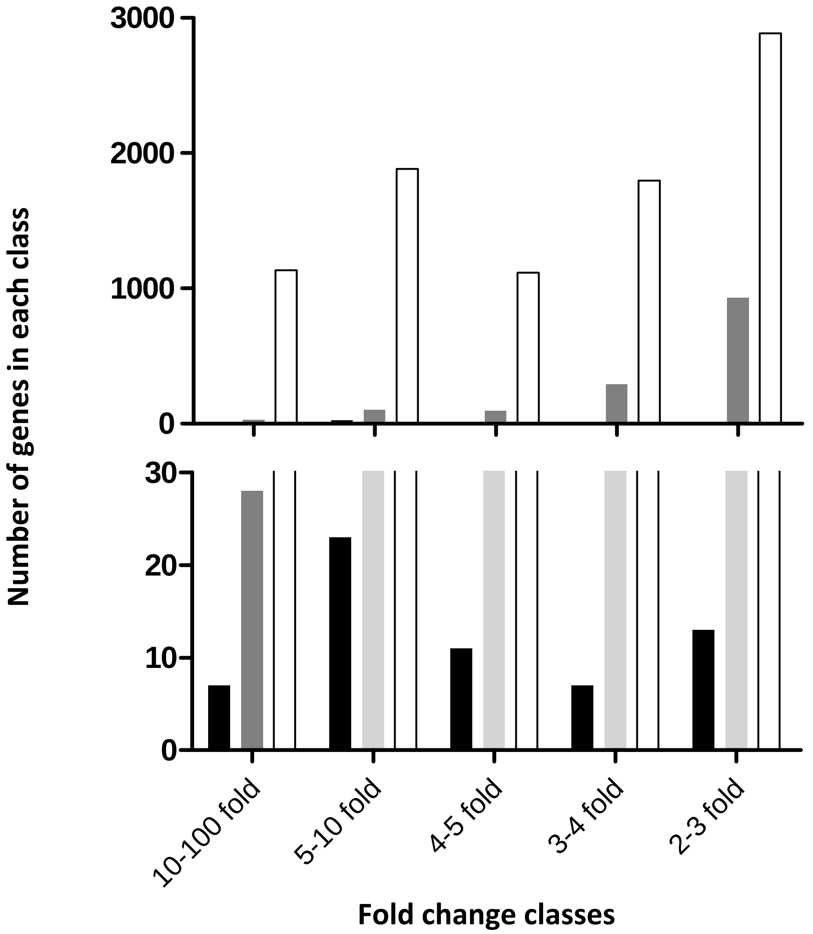

Additional data-6: Distribution of fold change magnitude by treatment for whole human genome transcript analysis. This figure represents the raw distribution of fold change magnitude for all transcripts that exhibited fold change ≥ 2.0 (p<0.01) in all three replicates of at least one treatment. CCM (white bars) caused significant change in 8,817 transcripts. DHA (dark bars) caused significant change in 61 transcripts. CCM+DHA (gray bars) caused significant change in 1,449 transcripts. Faded bars in the lower graph panel represent values that extend into the higher range illustrated in the upper graph panel. Upregulation (positive fold change) and downregulation (negative fold change) are represented as absolute values in this case, illustrating magnitude of change regardless of direction.
