# Supplementary material for: A synergistic antiproliferation effect of curcumin and docosahexaenoic acid in SK-BR-3 breast cancer cells: unique signaling not explained by the effects of either compound alone
Source: BMC Cancer. 2011 Apr 21;11:149. doi: 10.1186/1471-2407-11-149 (PMC3111403; doi:10.1186/1471-2407-11-149)
Supplement: Additional file 7 — Possible routes to phosphorylation of p53 and upregulated transcription of PPARγ. p53 and PPARγ were each used as single beginning nodes around which to build one-step expansion networks within MetaCore™ version 6.3 (GeneGo). [file 1471-2407-11-149-S7.PDF]

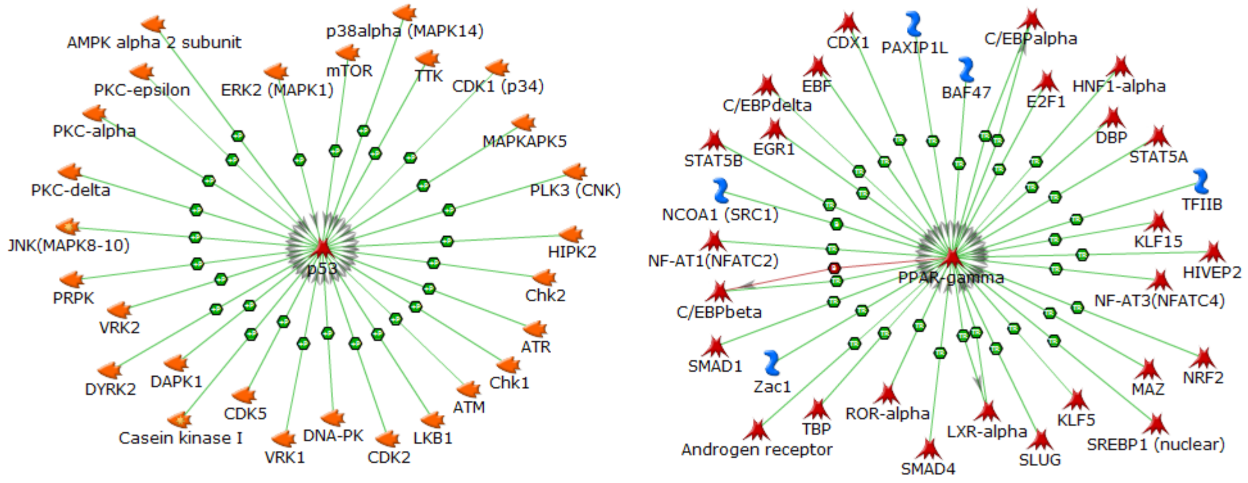


**Additional Data-7: Possible routes to phosphorylation of p53 and upregulated transcription of PPARγ.** p53 and PPARγ were each used as single beginning nodes around which to build one-step expansion networks within MetaCore™ version 6.3 (GeneGo). For p53, single step connections returned from the database were filtered for upstream connections, positive interaction, and phosphorylation. For PPARγ, single step connections returned from the database were filtered for upstream connections, positive interaction, and transcription regulation. Interaction database interrogation was limited to *Homo sapiens*.
