# Supplementary material for: A synergistic antiproliferation effect of curcumin and docosahexaenoic acid in SK-BR-3 breast cancer cells: unique signaling not explained by the effects of either compound alone
Source: BMC Cancer. 2011 Apr 21;11:149. doi: 10.1186/1471-2407-11-149 (PMC3111403; doi:10.1186/1471-2407-11-149)
Supplement: Additional file 8 — Literature annotation for transcript functional categories in Figure 3. Numbered references included in this file match reference numbers appearing to the right of each HGNC symbol in Figure 3. [file 1471-2407-11-149-S8.PDF]

Additional Data-8: Literature annotation for transcript functional categories in Figure 3.

As described in Methods, each transcript included in Figure 3 was assigned to a cancer-relevant functional category based upon literature annotation. Numbered references included below match reference numbers appearing to the right of each HGNC symbol in Figure 3.

Figure 3 gene annotation references

References

1. Li, B., et al., *Overload of the heat-shock protein H11/HspB8 triggers melanoma cell apoptosis through activation of transforming growth factor-beta-activated kinase 1.* Oncogene, 2007. **26**(24): p. 3521-3531.
2. Davis, R.J., *Signal transduction by the JNK group of MAP kinases.* Cell, 2000. **103**(2): p. 239-252.
3. Bruey, J.M., et al., *Bcl-2 and Bcl-X-L regulate proinf lammatory caspase-1 activation by interaction with NALP1.* Cell, 2007. **129**(1): p. 45-56.
4. Chu, Z.L., et al., *A novel enhancer of the Apaf1 apoptosome involved in cytochrome c-dependent caspase activation and apoptosis.* Journal of Biological Chemistry, 2001. **276**(12): p. 9239-9245.
5. Hlaing, T., et al., *Molecular cloning and characterization of DEFCAP-L and -S, two isoforms of a novel member of the mammalian Ced-4 family of apoptosis proteins.* Journal of Biological Chemistry, 2001. **276**(12): p. 9230-9238.
6. Liu, F., et al., *Expression of NALP 1 in cerebellar granule neurons stimulates apoptosis.* Cellular Signalling, 2004. **16**(9): p. 1013-1021.
7. Krumschnabel, G., et al., *The enigma of caspase-2: the laymen's view.* Cell Death and Differentiation, 2009. **16**(2): p. 195-207.
8. Ohiro, Y., et al., *A novel p53-inducible apoptogenic gene, PRG3, encodes a homologue of the apoptosis-inducing factor (AIF).* Febs Letters, 2002. **524**(1-3): p. 163-171.
9. Wu, M., et al., *AMID, an apoptosis-inducing factor-homologous mitochondrion-associated protein, induces caspase-independent apoptosis.* Journal of Biological Chemistry, 2002. **277**(28): p. 25617-25623.
10. Hitomi, J., et al., *Involvement of caspase-4 in endoplasmic reticulum stress-induced apoptosis and A beta-induced cell death.* Journal of Cell Biology, 2004. **165**(3): p. 347-356.
11. Kamada, S., et al., *Involvement of caspase-4(-like) protease in Fas-mediated apoptotic pathway.* Oncogene, 1997. **15**(3): p. 285-290.
12. Kim, S.J., et al., *Endoplasmic reticulum stress-induced caspase-4 activation mediates apoptosis and neurodegeneration in INCL.* Human Molecular Genetics, 2006. **15**(11): p. 1826-1834.
13. Faucheu, C., et al., *Identification of a cysteine protease closely related to interleukin-1 beta-converting enzyme.* European Journal of Biochemistry, 1996. **236**(1): p. 207-213.
14. Krippner-Heidenreich, A., et al., *Targeting of the transcription factor Max during apoptosis: phosphorylation-regulated cleavage by caspase-5 at an unusual glutamic acid residue in position P1.* Biochemical Journal, 2001. **358**: p. 705-715.
15. Martinon, F., K. Burns, and J. Tschopp, *The inflammasome: A molecular platform triggering activation of inflammatory caspases and processing of proIL-beta.* Molecular Cell, 2002. **10**(2): p. 417-426.

16. Munday, N.A., et al., *Molecular-cloning and pro-apoptotic activity of ICE(REL)II and ICE(REL)III, members of the ICE/CED-3 family of cysteine proteases.* Journal of Biological Chemistry, 1995. **270**(26): p. 15870-15876.
17. Kwon, J., et al., *Ubiquitin C-terminal hydrolase L-1 is essential for the early apoptotic wave of germinal cells and for sperm quality control during spermatogenesis.* Biology of Reproduction, 2005. **73**(1): p. 29-35.
18. Wang, W.J., et al., *Over-expression of ubiquitin carboxy terminal hydrolase-L1 induces apoptosis in breast cancer cells.* International Journal of Oncology, 2008. **33**(5): p. 1037-1045.
19. Liu, Y.C., et al., *Discovery of inhibitors that elucidate the role of UCH-L1 activity in the H1299 lung cancer cell line.* Chemistry & Biology, 2003. **10**(9): p. 837-846.
20. Fukutomi, S., et al., *Identification of methylation-silenced genes in colorectal cancer cell lines: Genomic screening using oligonucleotide arrays.* Scandinavian Journal of Gastroenterology, 2007. **42**(12): p. 1486-1494.
21. Tokumaru, Y., et al., *The role of PGP9.5 as a tumor suppressor gene in human cancer.* International Journal of Cancer, 2008. **123**(4): p. 753-759.
22. Yu, J., et al., *Epigenetic identification of ubiquitin carboxyl-terminal hydrolase L1 as a functional tumor suppressor and biomarker for hepatocellular carcinoma and other digestive tumors.* Hepatology, 2008. **48**(2): p. 508-518.
23. Foreman, J.E., et al., *Ligand activation of peroxisome proliferator-activated receptor-beta/delta (PPAR beta/delta) inhibits cell growth in a mouse mammary gland cancer cell line.* Cancer Letters. **288**(2): p. 219-225.
24. Naruhn, S., et al., *15-Hydroxyeicosatetraenoic Acid Is a Preferential Peroxisome Proliferator-Activated Receptor beta/delta Agonist.* Molecular Pharmacology. **77**(2): p. 171-184.
25. Berthier, A., et al., *High expression of gabarapl1 is associated with a better outcome for patients with lymph node-positive breast cancer.* British Journal of Cancer, 2010. **102**(6): p. 1024-1031.
26. Betin, V.M.S. and J.D. Lane, *Caspase cleavage of Atg4D stimulates GABARAP-L1 processing and triggers mitochondrial targeting and apoptosis.* Journal of Cell Science, 2009. **122**(14): p. 2554-2566.
27. Park, J.E., et al., *Positive regulation of apoptosis signal-regulating kinase 1 by dual-specificity phosphatase 13A.* Cellular and Molecular Life Sciences, 2010. **67**(15): p. 2619-2629.
28. Khalkhali-Ellis, Z., *Maspin: The new frontier.* Clinical Cancer Research, 2006. **12**(24): p. 7279-7283.
29. Sager, R., et al., *Maspin: A tumor suppressing serpin*, in *Attempts to Understand Metastasis Formation I*. 1996. p. 51-64.
30. Sheng, S.J., *The promise and challenge toward the clinical application of maspin in cancer.* Frontiers in Bioscience, 2004. **9**: p. 2733-2745.
31. Zou, Z.Q., et al., *Maspin, a serpin with tumor-suppressing activity in human mammary epithelial-cells.* Science, 1994. **263**(5146): p. 526-529.
32. Sheng, S., et al., *Maspin acts at the cell membrane to inhibit invasion and motility of mammary and prostatic cancer cells.* Proceedings of the National Academy of Sciences of the United States of America, 1996. **93**(21): p. 11669-11674.
33. Stark, A.M., et al., *Expression of metastasis suppressor gene maspin is reduced in breast cancer brain metastases and correlates with the estrogen receptor status.* Neurological Research, 2010. **32**(3): p. 303-308.
34. Khalkhali-Ellis, Z., et al., *Regulating the tumor suppressor gene maspin in breast cancer cells: A potential mechanism for the anticancer properties of tamoxifen.* Clinical Cancer Research, 2004. **10**(2): p. 449-454.
35. Liu, Z.S., et al., *Tamoxifen induces the expression of maspin through estrogen receptor-alpha.* Cancer Letters, 2004. **209**(1): p. 55-65.
36. Ben Shachar, B., et al., *The Tumor Suppressor Maspin Mediates E2F1-Induced Sensitivity of Cancer Cells to Chemotherapy.* Molecular Cancer Research, 2010. **8**(3): p. 363-372.

37. Zhou, Y.Q., et al., *Tumor suppressor function of BCSC-1 in nasopharyngeal carcinoma.* Cancer Science, 2009. **100**(10): p. 1817-1822.
38. Bowden, M.A., et al., *Serine proteases HTRA1 and HTRA3 are down-regulated with increasing grades of human endometrial cancer.* Gynecologic Oncology, 2006. **103**(1): p. 253-260.
39. Narklewicz, J., et al., *Changes in mRNA and protein levels of human HtrA1, HtrA2 and HtrA3 in ovarian cancer.* Clinical Biochemistry, 2008. **41**(7-8): p. 561-569.
40. Beleford, D., et al., *High Temperature Requirement A3 (HtrA3) Promotes Etoposide- and Cisplatin-induced Cytotoxicity in Lung Cancer Cell Lines.* Journal of Biological Chemistry. **285**(16): p. 12011-12027.
41. Lee, O.J., et al., *Hypermethylation and loss of expression of glutathione peroxidase-3 in Barrett's tumorigenesis.* Neoplasia, 2005. **7**(9): p. 854-861.
42. Yu, Y.P., et al., *Glutathione peroxidase 3, deleted or methylated in prostate cancer, suppresses prostate cancer growth and metastasis.* Cancer Research, 2007. **67**(17): p. 8043-8050.
43. Lai, C.P.K., J.F. Bechberger, and C.C. Naus, *Pannexin2 as a novel growth regulator in C6 glioma cells.* Oncogene, 2009. **28**(49): p. 4402-4408.
44. Lai, C.P.K., et al., *Tumor-suppressive effects of pannexin 1 in C6 glioma cells.* Cancer Research, 2007. **67**(4): p. 1545-1554.
45. Litvin, O., et al., *What is hidden in the pannexin treasure trove: the sneak peek and the guesswork.* Journal of Cellular and Molecular Medicine, 2006. **10**(3): p. 613-634.
46. El-Aziz, S.H.A., et al., *Cleavage of growth differentiation factor 15 (GDF15) by membrane type 1-matrix metalloproteinase abrogates GDF15-mediated suppression of tumor cell growth.* Cancer Science, 2007. **98**(9): p. 1330-1335.
47. Park, J.Y., et al., *Expression of nonsteroidal anti-inflammatory drug-activated gene-1 (NAG-1) inversely correlates with tumor progression in gastric adenomas and carcinomas.* Journal of Cancer Research and Clinical Oncology, 2008. **134**(9): p. 1029-1035.
48. Kim, J.S., et al., *The conventional nonsteroidal anti-inflammatory drug sulindac sulfide arrests ovarian cancer cell growth via the expression of NAG-1/MIC-1/GDF-15.* Molecular Cancer Therapeutics, 2005. **4**(3): p. 487-493.
49. Fang, D., et al., *Expression of microtubule-associated protein 2 in benign and malignant melanocytes - Implications for differentiation and progression of cutaneous melanoma.* American Journal of Pathology, 2001. **158**(6): p. 2107-2115.
50. Soltani, M.H., et al., *Microtubule-associated protein 2, a marker of neuronal differentiation, induces mitotic defects, inhibits growth of melanoma cells, and predicts metastatic potential of cutaneous melanoma.* American Journal of Pathology, 2005. **166**(6): p. 1841-1850.
51. Bhat, K.M.R. and V. Setaluri, *Microtubule-associated proteins as targets in cancer chemotherapy.* Clinical Cancer Research, 2007. **13**(10): p. 2849-2854.
52. Androutsopoulos, V.P., et al., *CYP1-mediated antiproliferative activity of dietary flavonoids in MDA-MB-468 breast cancer cells.* Toxicology, 2009. **264**(3): p. 162-170.
53. Chua, M.S., et al., *Role of CYP1A1 in modulation of antitumor properties of the novel agent 2-(4-amino-3-methylphenyl)benzothiazole (DF 203, NSC 674495) in human breast cancer cells.* Cancer Research, 2000. **60**(18): p. 5196-5203.
54. Mense, S.M., J. Chhabra, and H.K. Bhat, *Preferential induction of cytochrome P450 1A1 over cytochrome P4501B1 in human breast epithelial cells following exposure to quercetin.* Journal of Steroid Biochemistry and Molecular Biology, 2008. **110**(1-2): p. 157-162.
55. Murray, G.I., *The role of cytochrome P450 in tumour development and progression and its potential in therapy.* Journal of Pathology, 2000. **192**(4): p. 419-426.
56. Androutsopoulos, V.P., et al., *Anticancer effects of the flavonoid diosmetin on cell cycle progression and proliferation of MDA-MB 468 breast cancer cells due to CYP1 activation.* Oncology Reports, 2009. **21**(6): p. 1525-1528.

57. Ware, W.R., *Nutrition and the Prevention and Treatment of Cancer: Association of Cytochrome P450 CYP1B1 With the Role of Fruit and Fruit Extracts.* Integrative Cancer Therapies, 2009. **8**(1): p. 22-28.
58. Ware, W.R., *P450 CYP1B1 mediated fluorescent tumor markers: A potentially useful approach for photodynamic therapy, diagnosis and establishing surgical margins.* Medical Hypotheses, 2009. **72**(1): p. 67-70.
59. Yadav, S., et al., *Association of single nucleotide polymorphisms in CYP1B1 and COMT genes with breast cancer susceptibility in Indian women.* Disease Markers, 2009. **27**(5): p. 203-210.
60. Li, M., et al., *Aberrant expression of zinc transporter ZIP4 (SLC39A4) significantly contributes to human pancreatic cancer pathogenesis and progression.* Proceedings of the National Academy of Sciences of the United States of America, 2007. **104**(47): p. 18636-18641.
61. Nishihara, H., et al., *DOCK2 associates with CrkL and regulates Rac1 in human leukemia cell lines.* Blood, 2002. **100**(12): p. 3968-3974.
62. Wang, L., et al., *DOCK2 regulates cell proliferation through Rac and ERK activation in B cell lymphoma.* Biochemical and Biophysical Research Communications, 2010. **395**(1): p. 111-115.
63. Murooka, T.T., R. Rahbar, and E.N. Fish, *CCL5 promotes proliferation of MCF-7 cells through mTOR-dependent mRNA translation.* Biochemical and Biophysical Research Communications, 2009. **387**(2): p. 381-386.
64. Chuang, J.Y., et al., *CCL5/CCR5 Axis Promotes the Motility of Human Oral Cancer Cells.* Journal of Cellular Physiology, 2009. **220**(2): p. 418-426.
65. Laubli, H., K.S. Spanaus, and L. Borsig, *Selectin-mediated activation of endothelial cells induces expression of CCL5 and promotes metastasis through recruitment of monocytes.* Blood, 2009. **114**(20): p. 4583-4591.
66. Pinilla, S., et al., *Tissue resident stem cells produce CCL5 under the influence of cancer cells and thereby promote breast cancer cell invasion.* Cancer Letters, 2009. **284**(1): p. 80-85.
67. Soria, G., et al., *Concomitant expression of the chemokines RANTES and MCP-1 in human breast cancer: A basis for tumor-promoting interactions.* Cytokine, 2008. **44**(1): p. 191-200.
68. Xu, W.T., et al., *Human mesenchymal stem cells (hMSCs) target osteosarcoma and promote its growth and pulmonary metastasis.* Cancer Letters, 2009. **281**(1): p. 32-41.
69. Goel, A. and R. Janknecht, *Concerted activation of ETS protein ER81 by p160 coactivators, the acetyltransferase p300 and the receptor tyrosine kinase HER2/Neu.* Journal of Biological Chemistry, 2004. **279**(15): p. 14909-14916.
70. Jane-Valbuena, J., et al., *An Oncogenic Role for ETV1 in Melanoma.* Cancer Research, 2010. **70**(5): p. 2075-2084.
71. Shin, S., et al., *Rcl Is a Novel ETV1/ER81 Target Gene Upregulated in Breast Tumors.* Journal of Cellular Biochemistry, 2008. **105**(3): p. 866-874.
72. Shin, S., et al., *Induction of Prostatic Intraepithelial Neoplasia and Modulation of Androgen Receptor by ETS Variant 1/ETS-Related Protein 81.* Cancer Research, 2009. **69**(20): p. 8102-8110.
73. Finger, F.P., *Reining in cytokinesis with a septin corral.* Bioessays, 2005. **27**(1): p. 5-8.
74. Joo, E., C.W. Tsang, and W.S. Trimble, *Septins: Traffic control at the cytokinesis intersection.* Traffic, 2005. **6**(8): p. 626-634.
75. Feng, C.J., et al., *Expression of Mcm7 and Cdc6 in Oral Squamous Cell Carcinoma and Precancerous Lesions.* Anticancer Research, 2008. **28**(6A): p. 3763-3769.
76. Ren, B., et al., *MCM7 amplification and overexpression are associated with prostate cancer progression.* Oncogene, 2006. **25**(7): p. 1090-1098.
77. Yoshida, K. and I. Inoue, *Conditional expression of MCM7 increases tumor growth without altering DNA replication activity.* Febs Letters, 2003. **553**(1-2): p. 213-217.
78. Hartwell, K.A., et al., *The Spemann organizer gene, Goosecoid, promotes tumor metastasis.* Proceedings of the National Academy of Sciences of the United States of America, 2006. **103**(50): p. 18969-18974.

79. Bachmeier, B.E., et al., *Curcumin downregulates the inflammatory cytokines CXCL1 and-2 in breast cancer cells via NF kappa B.* Carcinogenesis, 2008. **29**(4): p. 779-789.
80. Kawanishi, H., et al., *Secreted CXCL1 is a potential mediator and marker of the tumor invasion of bladder cancer.* Clinical Cancer Research, 2008. **14**(9): p. 2579-2587.
81. Warner, K.A., et al., *Endothelial cells enhance tumor cell invasion through a crosstalk mediated by CXC chemokine signaling.* Neoplasia, 2008. **10**(2): p. 131-139.
82. Wang, B., et al., *A Key Role for Early Growth Response-1 and Nuclear Factor-kappa B in Mediating and Maintaining GRO/CXCR2 Proliferative Signaling in Esophageal Cancer.* Molecular Cancer Research, 2009. **7**(5): p. 755-764.
83. Wang, D.Z., et al., *CXCL1 induced by prostaglandin E-2 promotes angiogenesis in colorectal cancer.* Journal of Experimental Medicine, 2006. **203**(4): p. 941-951.
84. Fernandis, A.Z., et al., *Regulation of CXCR4-mediated chemotaxis and chemoinvasion of breast cancer cells.* Oncogene, 2004. **23**(1): p. 157-167.
85. Helbig, G., et al., *NF-kappa B promotes breast cancer cell migration and metastasis by inducing the expression of the chemokine receptor CXCR4.* Journal of Biological Chemistry, 2003. **278**(24): p. 21631-21638.
86. Muller, A., et al., *Involvement of chemokine receptors in breast cancer metastasis.* Nature, 2001. **410**(6824): p. 50-56.
87. Acs, G., et al., *Erythropoietin and erythropoietin receptor expression in human cancer.* Cancer Research, 2001. **61**(9): p. 3561-3565.
88. Jeong, J.Y., et al., *Characterization of erythropoietin receptor and erythropoietin expression and function in human ovarian cancer cells.* International Journal of Cancer, 2008. **122**(2): p. 274-280.
89. Solar, P., et al., *Erythropoietin treatment of human ovarian cancer cells results in enhanced signaling and a paclitaxel-resistant phenotype.* International Journal of Cancer, 2008. **122**(2): p. 281-288.
90. Ghezzi, P. and M. Brines, *Erythropoietin as an antiapoptotic, tissue-protective cytokine.* Cell Death and Differentiation, 2004. **11**: p. S37-S44.
91. Um, M. and H.F. Lodish, *Antiapoptotic effects of erythropoietin in differentiated neuroblastoma SH-SY5Y cells require activation of both the STAT5 and AKT signaling pathways.* Journal of Biological Chemistry, 2006. **281**(9): p. 5648-5656.
92. Vairano, M., et al., *Erythropoietin exerts anti-apoptotic effects on rat microglial cells in vitro.* European Journal of Neuroscience, 2002. **16**(4): p. 584-592.
93. Wenker, S.D., et al., *Differential Antiapoptotic Effect of Erythropoietin on Undifferentiated and Retinoic Acid-Differentiated SH-SY5Y Cells.* Journal of Cellular Biochemistry, 2010. **110**(1): p. 151-161.
94. Sytkowski, A.J., *Does Erythropoietin Have a Dark Side? Epo Signaling and Cancer Cells.* Sci. STKE, 2007. **2007**(395): p. pe38-.
95. Li, C.M., et al., *PEG10 is a c-MYC target gene in cancer cells.* Cancer Research, 2006. **66**(2): p. 665-672.
96. Okabe, H., et al., *Involvement of PEG10 in human hepatocellular carcinogenesis through interaction with SIAH1.* Cancer Research, 2003. **63**(12): p. 3043-3048.
97. Tsou, A.P., et al., *Overexpression of a novel imprinted gene, PEG10, in human hepatocellular carcinoma and in regenerating mouse livers.* Journal of Biomedical Science, 2003. **10**(6): p. 625-635.
