# Supplementary material for: A synergistic antiproliferation effect of curcumin and docosahexaenoic acid in SK-BR-3 breast cancer cells: unique signaling not explained by the effects of either compound alone
Source: BMC Cancer. 2011 Apr 21;11:149. doi: 10.1186/1471-2407-11-149 (PMC3111403; doi:10.1186/1471-2407-11-149)
Supplement: Additional file 9 — Data represented by Figure 3. Fold change values and associated P-values for transcripts represented in Figure 3. [file 1471-2407-11-149-S9.PDF]

Additional Data -9

Fold change values and associated P-values for transcripts represented in Figure 3.

Apoptosis

Gene ID	CCM fold change	P-value	DHA fold change	P-value	CCM+DHA fold change	P-value
NLRP1	-1.106	7.04E-01	1.592	4.83E-01	15.502	0.00E+00
DUSP13	1.783	1.53E-09	1.045	6.22E-01	12.212	0.00E+00
UCHL1	1.267	8.31E-02	-1.142	2.23E-01	11.058	4.07E-40
HSPB8	4.517	5.98E-35	1.124	8.08E-02	6.195	2.06E-44
GABARAPL1	2.756	7.58E-21	-1.051	5.88E-01	5.651	4.67E-46
AIFM2	1.075	4.76E-01	1.024	7.82E-01	4.111	1.79E-35
ANGPTL4	1.025	7.75E-01	5.789	1.50E-41	4.051	9.80E-33
CASP5	1.366	1.08E-03	1.017	8.52E-01	2.918	5.58E-21
CASP4	1.176	1.19E-01	1.003	8.76E-01	2.873	2.79E-22

Tumor suppression

Gene ID	CCM fold change	P-value	DHA fold change	P-value	CCM+DHA fold change	P-value
SERPINB5	3.326	1.10E-02	1.131	6.73E-01	18.895	8.58E-20
HTRA3	40.675	1.35E-44	1.215	8.04E-01	12.724	7.77E-07
VWA5A	-1.079	8.01E-01	1.098	7.97E-01	7.898	1.10E-04
GPX3	2.127	2.26E-13	1.136	2.16E-01	6.467	6.40E-41
PANX2	-1.569	3.13E-01	1.358	5.20E-01	5.858	1.90E-04
GDF15	2.507	1.38E-19	1.734	9.22E-10	2.979	7.67E-26

Anti-metastasis

Gene ID	CCM fold change	P-value	DHA fold change	P-value	CCM+DHA fold change	P-value
MAP2	2.425	6.01E-04	-1.007	3.97E-01	8.964	1.55E-24

Small molecule enhancement

Gene ID	CCM fold change	P-value	DHA fold change	P-value	CCM+DHA fold change	P-value
CYP1A1	1.734	4.60E-06	-1.252	8.14E-02	100.000	0.00E+00
CYP1A2	-1.436	5.00E-01	-1.406	5.12E-01	29.491	2.11E-17
CYP1B1	-2.422	2.09E-15	1.116	2.30E-01	7.415	1.73E-44

Tumor progression/growth

Gene ID	CCM fold change	P-value	DHA fold change	P-value	CCM+DHA fold change	P-value
DOCK2	-10.776	8.16E-31	1.041	5.58E-01	-6.784	2.96E-24
ETV1	5.455	2.02E-16	-1.540	2.03E-01	-5.068	1.52E-03
CCL5	-2.390	1.39E-03	-1.158	3.36E-01	-5.011	6.53E-04
SLC39A4	-2.356	2.72E-03	-1.496	2.26E-01	-4.698	1.30E-04
CCL2	-1.644	1.19E-42	1.327	1.03E-14	-2.592	0.00E+00

Cell cycle progression

Gene ID	CCM fold change	P-value	DHA fold change	P-value	CCM+DHA fold change	P-value
MCM7	-1.849	3.79E-10	-1.036	6.83E-01	-6.635	1.53E-42
SEPT6	-2.463	6.90E-04	1.222	5.32E-01	-2.951	2.48E-23

Metastasis

Gene ID	CCM fold change	P-value	DHA fold change	P-value	CCM+DHA fold change	P-value
GSC	-2.948	1.58E-01	-10.840	2.67E-04	-8.974	6.73E-04
CXCL1	1.535	3.67E-03	-1.091	1.85E-01	-7.711	5.04E-08
CXCR4	1.292	1.05E-08	1.011	6.55E-01	-2.947	0.00E+00

Anti-apoptosis/cell survival

Gene ID	CCM fold change	P-value	DHA fold change	P-value	CCM+DHA fold change	P-value
EPO	1.475	2.32E-03	1.367	8.95E-03	-8.722	6.17E-21
PEG10	1.818	5.03E-09	-1.316	1.45E-02	-5.689	3.51E-39

All fold change values and associated p-values were produced according to the workflow described in the Methods section *Processing of preselected candidate gene lists and gene ratio lists from MBGS.* Data and conditional statements used to produce these values may be seen in these Supplementary Data files:

Preselected UNTvsZadd all.xlsx
Preselected UNTvsCCM all.xlsx
Preselected UNTvsDHA all.xlsx

ALL 3 rep responders for CCM.xlsx
Extracting ALL responders that are not CCM responders.xlsx
Extracting CCM response values for ALL that are not CCM.xlsx

ALL 3 rep responders for DHA.xlsx
Extracting ALL responders that are not DHA responders.xlsx
Extracting CCM response values for ALL that are not DHA.xlsx
